# The Transition from Proliferation to Differentiation in Colorectal Cancer Is Regulated by the Calcium Activated Chloride Channel A1

**DOI:** 10.1371/journal.pone.0060861

**Published:** 2013-04-12

**Authors:** Bo Yang, Lin Cao, Bin Liu, Colin D. McCaig, Jin Pu

**Affiliations:** 1 Department of General Surgery, The 309th Hospital of PLA, Beijing, China; 2 School of Medical Sciences, Institute of Medical Sciences, University of Aberdeen, Aberdeen, Scotland, United Kingdom; Sapporo Medical University, Japan

## Abstract

Breaking the balance between proliferation and differentiation in animal cells can lead to cancer, but the mechanisms maintaining this balance remain largely undefined. The calcium activated chloride channel A1 (CLCA1) is a member of the calcium sensitive chloride conductance family of proteins and is expressed mainly in the colon, small intestine and appendix. We show that CLCA1 plays a functional role in differentiation and proliferation of Caco-2 cells and of intestinal tissue. Caco-2 cells spontaneously differentiate either in confluent culture or when treated with butyrate, a molecule present naturally in the diet. Here, we compared CLCA1 expressional levels between patients with and without colorectal cancer (CRC) and determined the functional role of CLCA1 in differentiation and proliferation of Caco-2 cells. We showed that: 1) CLCA1 and CLCA4 expression were down-regulated significantly in CRC patients; 2) CLCA1 expression was up-regulated in Caco-2 cells induced to differentiate by confluent culture or by treatment with sodium butyrate (NaBT); 3) Knockdown of CLCA1 with siRNA significantly inhibited cell differentiation and promoted cell proliferation in Caco-2 confluent cultures, and 4) In Caco-2 3D culture, suppression of CLCA1 significantly increased cell proliferation and compromised NaBT-induced inhibition of proliferation. In conclusion, CLCA1 may contribute to promoting spontaneous differentiation and reducing proliferation of Caco-2 cells and may be a target of NaBT-induced inhibition of proliferation and therefore a potential diagnostic marker for CRC prognosis.

## Introduction

In mammalian intestine the enterocytes are renewed continually every 4–8 days. This occurs through a coordinated series of events involving proliferation, differentiation, and migration from the intestinal crypts upwards toward the lumen [1]. Disruption of the balance between cell division and differentiation can lead to disease states such as cancer [2]. Alterations of the tightly regulated balance between the highly proliferative/less differentiated enterocytes and the non-proliferative/highly differentiated state may lead to hyperplasia, benign (polyps) or malignant tumors [3]. The Wnt signaling pathway is the primary mechanism controlling proliferation of enterocytes in the intestinal crypts [4].

Ion channels contribute to tumors by regulating the basic cellular processes of proliferation, differentiation, and apoptosis [5]. For example, KCNK9 (potassium channel subfamily K member 9) is overexpressed and contributes to tumorigenesis by promoting cancer cell survival in breast cancer [6]. GIRK1 (G-protein inwardly rectifying potassium channel 1) expression increased in 50/72 non-small-cell lung cancer patients and the presence of this mRNA was associated with significantly reduced five-year survival rates [7]. In addition, the TRPM8 channel protein (Transient receptor potential cation channel subfamily M member 8) a prostate-specific marker was up-regulated in tumor tissue [8]. Studies describing the occurrence of individual ion channels in tumor cells and their functional consequences on growth, migration, or invasion are increasing [9].

In the colon, chloride channels (CLCs) form a large functional family with structurally diverse members that play an important role in the regulation of epithelial fluid and electrolyte transport [10]. Activation of the chloride current through specialized volume-regulated anion channels (VRACs) in response to cell swelling (I_Cl, swell_) is one of the major mechanisms by which cells restore their volume following hypo-osmotic stress (RVD) [11]. This is important since there is a direct link between apoptotic resistance conferred by the antiapoptotic Bcl-2 protein and the strengthening of RVD capability due to up-regulation of I_Cl, swell_ [12]. In addition, the Chloride channel 3 (CLC3) protein is among the prostate-specific VRACs and is up-regulated in androgen-independent prostate cancer cells [13]. Calcium-activated chloride channels (CLCAs) also are expressed in bovine [14], mouse [15] and human [16] enterocytes and there is an inverse correlation between the levels of chloride channel (CLCA1 and CLCA2) expression and tumorigenicity, indicating that they act as suppressors of breast and colorectal cancer [17], [18], [19]. However, the detailed biological function of CLCAs remains to be determined. Therefore, we investigated whether CLCA1 contributes to tumorigenesis by regulating the balance between proliferation and differentiation in enterocytes.

The human intestinal cancer cell line Caco-2 is a well-established model system to study cellular differentiation of human enterocytes since it differentiates spontaneously into polarized cells with morphological and biochemical features of small intestinal enterocytes [20]. Additionally, Caco-2 cells also differentiate when exposed to the physiologically relevant short-chain fatty acid, butyrate [19]. Short chain fatty acids (SCFA), principally butyrate, propionate and acetate, are produced in the gut through the fermentation of dietary fiber by the colonic microbiota [21]. Butyrate in particular is the preferred energy source for the cells of the colonic mucosa and exerts various biological effects on cultured mammalian cells, including inhibition of cell proliferation, apoptosis and induction of differentiation [22], [23]. Because of this, its therapeutic potential in colon cancer has been proposed [23]. Caco-2 cells, when grown as a confluent monolayer or exposed to sodium butyrate (NaBT) treatment, differentiate to mimic phenotypically and functionally mature colonic epithelium and are a useful model for investigating the molecular mechanisms of differentiation in CRC. Here, we demonstrate that CLCA1 (Calcium-activated chloride channel family member 1) plays a functional role in regulating the differentiation and proliferation of Caco-2 cells.

## Results

### Down-regulation of CLCA1 Expression in Colorectal cancer (CRC) Patients

**Figure 1:**
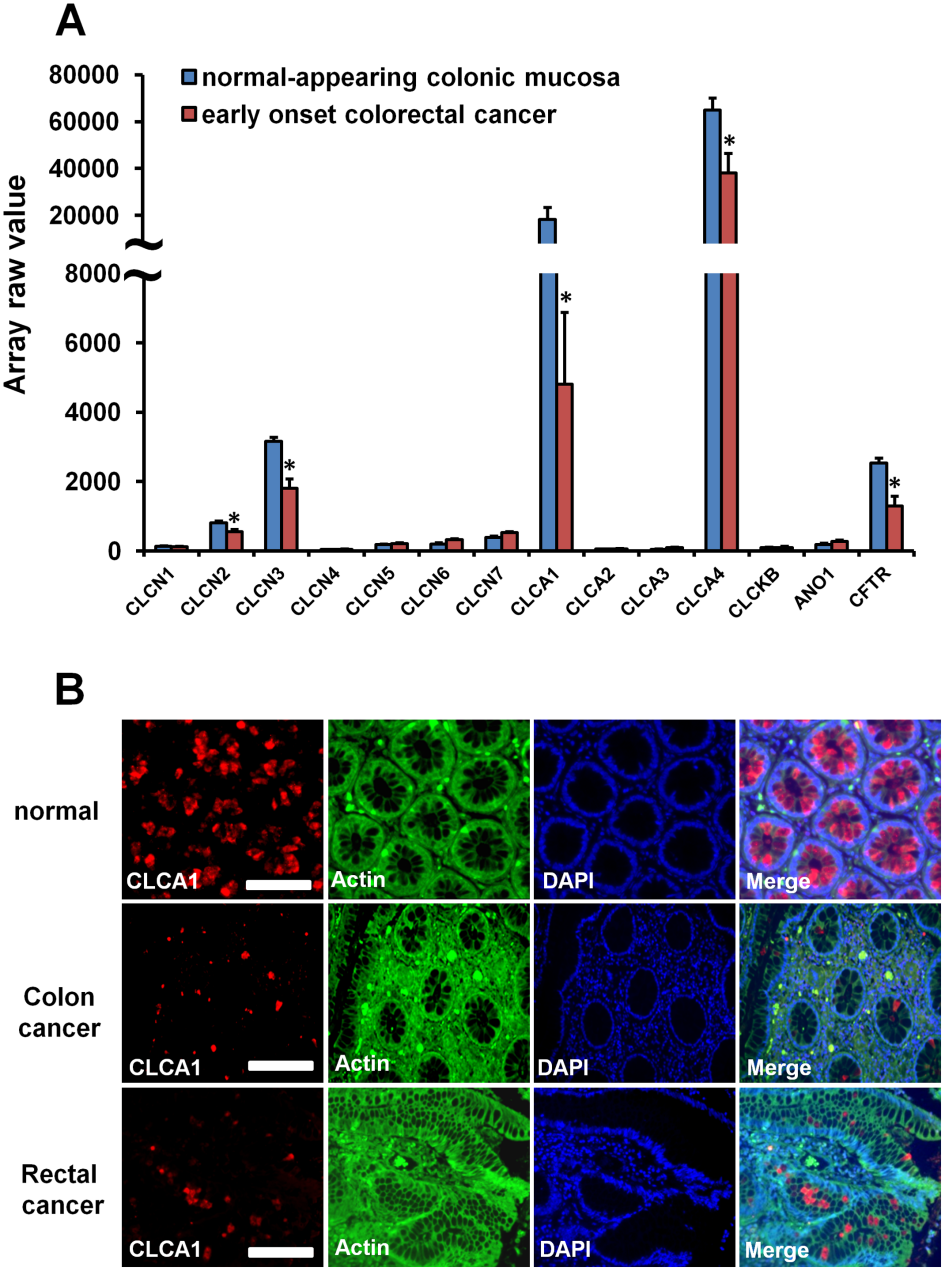
Down-regulation of CLCA1 expression in CRC. **A.** Normalized raw expression values of chloride channel family members were analyzed in human colonic mucosa and early CRC tissues from Gene Expression Omnibus (reference series is GSE4017). CLCA1, CLCA4, CLCN2, CLCN3 and CFTR were down-regulated significantly in early CRC patients. Values are mean±s.e.m, *p<0.01. **B.** Analysis of immunofluorescence demonstrated that the expression of CLCA1 was reduced in CRC tissues in contrast to normal colonic mucosa. Bar = 50 µm.

Firstly, we investigated the expression level in humans of chloride channels in normal and tumour tissues using the microarray database of NCBI (http://www.ncbi.nlm.nih.gov/geo/). The expression levels of CLCN2, CLCN3, CLCA1, CLCA4 and CFTR in early CRC patient were significantly reduced 31%, 59%, 74%, 41% and 58% respectively compared to normal colonic mucosa (Fig. 1A). To further confirm the down-regulation of CLCA1 in CRC patients, we used immunofluorescent staining to detect CLCA1 expression in both CRC and normal intestinal tissues and found the expression of CLCA1 reduced significantly in CRC intestinal tissues (Fig. 1B). One of the features in tumorigenesis is the high proliferation/low differentiation rate of cells. Perhaps CLCA1 contributes to tumorigenesis by regulating the balance between proliferation and differentiation in enterocytes.

### Up-regulation of CLCA1 Induced by Sodium Butyrate in Caco-2 Cells

**Figure 2:**
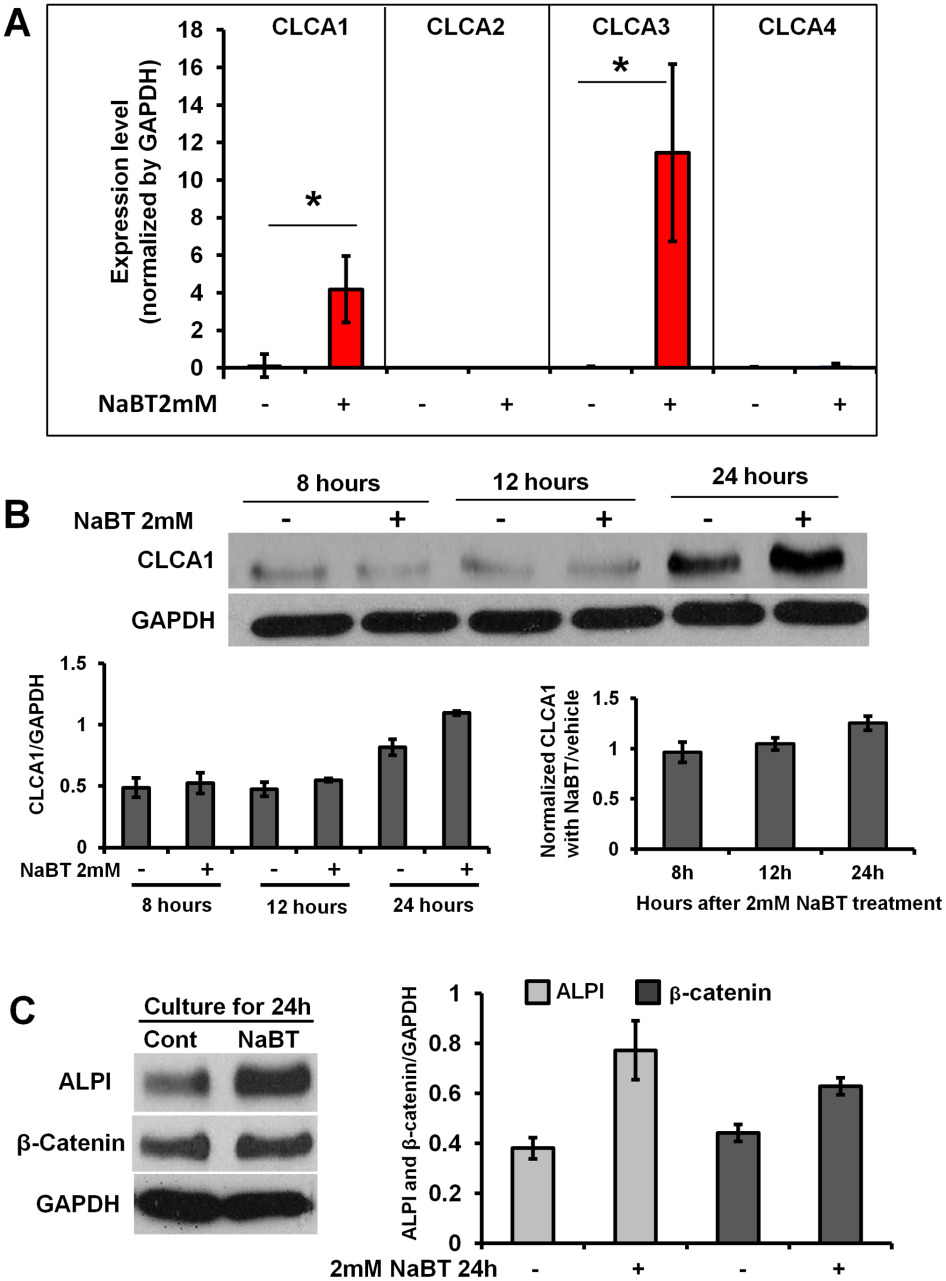
Sodium Butyrate (NaBT) up-regulated CLCA1 expression and promoted differentiation in Caco-2 cells. **A.** The mRNA expression of CLCA1, A2, A3 and A4 were measured using quantitative RT-PCR. CLCA1 and CLCA3 were up-regulated respectively in Caco-2 cells after treatment with 2 mM NaBT for 24 hours. Data are presented as mean ± s.e.m from three independent experiments, *p<0.01. **B.** Caco-2 monolayer was cultured for different time periods with/without 2 mM NaBT treatment. Expression of CLCA1 was detected by western blot. In 8 and 12 hours confluent culture, neither control nor NaBT increased the expression of CLCA1. When Caco-2 monolayers were cultured for 24 hours, both control and NaBT up-regulated CLCA1 expression, but NaBT enhanced CLCA1 expression much more than was seen in controls. **C.** NaBT elevated ALPI and β-catenin expression (differentiation markers) in confluent cultures of Caco-2 cells. The histograms in B and C show the relative intensity of CLCA1 90 KD, ALPI and β-catenin expressed as a ratio with respect to the GAPDH control. All results were from three independent experiments.

A pro-differentiating effect of sodium butyrate (NaBT) has been studied extensively in various malignant cell lines [24], [25]. We analyzed Ca^2+^–dependent chloride channel expression patterns in Caco-2 cells using quantitative RT-PCR assay (qPCR). We found that expression levels of CLCA1 and CLCA3 were upregulated 35-fold and over 100-fold respectively after 2 mM NaBT treatment for 24 hours (Fig. 2A). Western blots additionally showed that the CLCA1 protein increase was time-dependent. When Caco-2 cells were treated with NaBT for 24 hours, CLCA1 expression was upregulated significantly compared to the no treatment group (Fig. 2B). However, there was little or no expression of CLCA1 following shorter exposure (8 and 12 hours treatments, Fig. 2B). Expression of CLCA1 in monolayers not treated with NaBT also displayed a significant increase at 24 hours (compared to 8 and 12 hours cultures) due to the spontaneous differentiation of Caco-2 cells in confluent cultures (Fig. 2B). NaBT also elevated the expression of intestinal alkaline phosphatase (ALPI) and β-catenin in Caco-2 cells (Fig. 2C). Both are markers of enterocyte differentiation and are upregulated in differentiated cells. Our data suggest that CLCA1 may mediate the cell differentiation induced by confluent culture and by NaBT in Caco-2 cells.

### CLCA1 is Upregulated at an Early Stage during Spontaneous Differentiation of Caco-2 Cells in Confluent Culture

**Figure 3:**
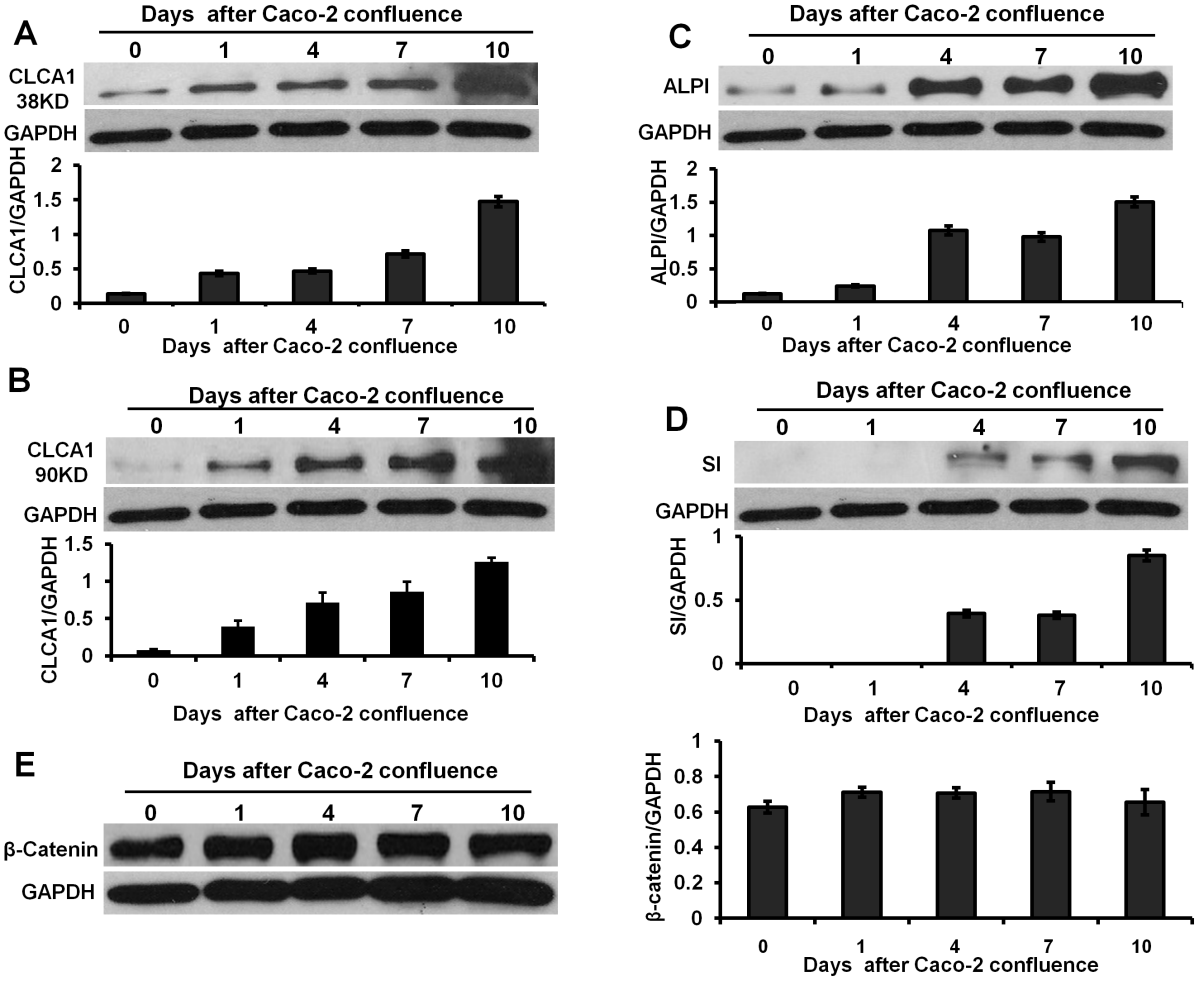
Expression of CLCA1, ALPI and sucrase-isomaltase was upregulated during spontaneous differentiation of Caco-2 monolayer. **A and B.** Expression of CLCA1 subunits (38 KD and 90 KD) were up-regulated after 24 hour of confluent culture and reached a peak at 10 days of culture. **C.** ALPI as a marker of Caco-2 cell differentiation was up-regulated significantly after 4 days of confluent culture. **D.** Expression of sucrase-isomaltase (SI), another cell differentiation marker, also was increased significantly after 4 days of confluent culture. **E.** Expression of β-catenin was enhanced slightly over time in culture. The histograms in A to E show the relative intensity of CLCA1, ALPI, SI and β-catenin expressed as a ratio with respect to the GAPDH control. All results were analyzed from three independent experiments.

**Figure 4:**
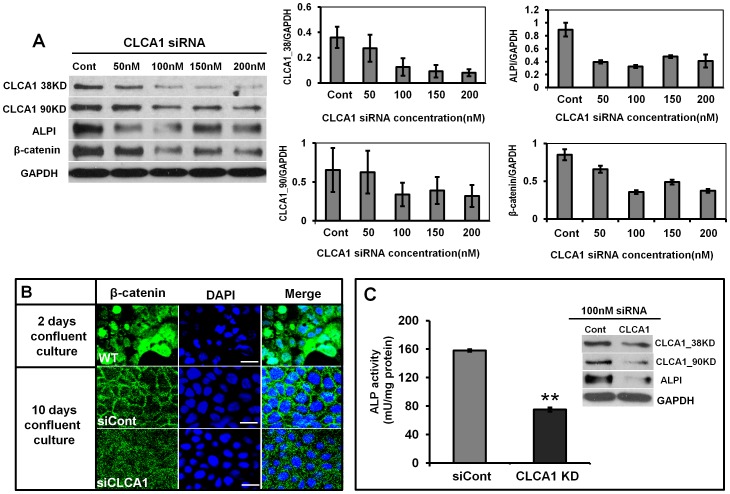
CLCA1 is required for spontaneous differentiation in Caco-2 cells. **A.** Caco-2 cells were transfected transiently with 0, 50, 100, 150 and 200 nM siRNA^clca1^ and blotted for CLCA1. siRNA^clca1^ at 100 nM or above effectively inhibited CLCA1 and downregulated expression of ALPI and β-catenin. **B.** Immunofluorescent staining showed the expression of β-catenin in confluent cultures of Caco-2 cells. β-catenin was located mainly in the nucleus of the cells at early stages of culture (2 days). After 10 days culture, β-catenin had translocated to the cell membrane. Knockdown of CLCA1 reduced distribution of β-catenin on the membrane. **C.** Caco-2 cells were treated with either siRNA negative control or siRNA^clca1^ and then were cultured for 72 hours. Cell lysates were collected for detection of ALP activity using the Alkaline Phosphatase Detection Kit or for ALP expression by western blot. The result shows that both ALP activity and expression were reduced significantly in CLCA1 knockdown cells. The histograms in A showed the relative intensity of CLCA1 38 KD, 90 KD, ALPI and β-catenin expressed as a ratio with respect to the GAPDH control. All results were from three independent experiments. **p<0.01.

Culture to confluence induces the spontaneous differentiation of Caco-2 cells [19], [24], [25], [26]. Using this model, we asked whether CLCA1 contributed to the differentiation of Caco-2 cells. Firstly, we detected the expression of CLCA1 and the two differentiation markers ALPI and sucrase-isomaltase (SI) in confluent culture. We found that expression of CLCA1 (including the two different cell-surface-associated subunits 38 KD and 90 KD [16]) was detected at very low levels at the beginning of the culture. However, as cultures became confluent, CLCA1 expression started to increase in a time dependent manner. This increase in expression was evident within 1 day (Fig. 3A, 3B). Expression of ALPI and SI also showed a significant time dependent up-regulation, but this did not occur until day 4, later than for CLCA1 expression (Fig. 3C, 3D). In addition, we detected also the expression of β-catenin at different days of Caco-2 cell confluent culture. We found that the β-catenin increased slightly in Caco-2 confluent monolayer (Fig. 3E). Subcellular fractionation studies showed that the increase of β-catenin was attributable to an increase in the membrane fraction of β-catenin, whereas cytosolic levels remained unchanged (Fig. 4B) [27]. These data suggest that the expression of CLCA1 may contribute to the spontaneous differentiation of Caco-2 cells.

### CLCA1 is Required for Spontaneous Differentiation of Caco-2 Cells

To determine the functional role of CLCA1 in Caco-2 cell differentiation, we used a stealth short-interfering RNA (siRNA^clca1^) to knock-down the expression of CLCA1 in Caco-2 cells. After 72 hr transfection, cells were tested for expression of CLCA1, ALPI and β-catenin by western blot (Fig. 4A). Our data showed that CLCA1 expression was downregulated in a dose-dependent manner in response to increasing transfection concentrations of siRNA^clca1^. To avoid off-target effects by higher concentration of siRNA, we chose 100 nM siRNA^clca1^ as an optimal concentration for subsequent experiments.

ALPI and β-catenin expression were reduced significantly upon CLCA1 knockdown. Moreover, confocal imaging showed a reduced cell membrane staining of β-catenin in siRNA_clca1_ knockdown cells (Fig. 4B). To further confirm the pro-differentiation role of CLCA1 in Caco-2 cells, we detected ALP activity using a cell differentiation detection kit in Caco-2 cells. Our data demonstrated that ALP activity was inhibited significantly in CLCA1 knockdown cells (Fig. 4C). These results indicated that the CLCA1 plays a key role in regulation of spontaneous differentiation of Caco-2 cells.

### CLCA1 Plays an Anti-proliferative Role in Caco-2 Cells

**Figure 5:**
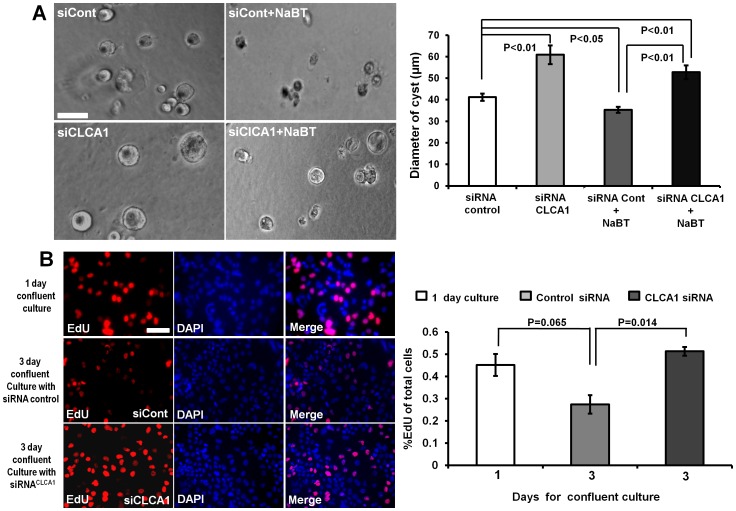
The effect of CLCA1 on proliferation of enterocytes. **A.** Caco-2 cells either treated by siRNA negative control, or with CLCA1 siRNA alone, or with added NaBT were seeded in the 25% Matrigel and kept in 5% CO_2_ and 37°C for 5 days. The diameter of cysts was measured and analyzed using Metamoph software. The mean colony size in Caco-2 CLCA1 knockdown cells increased significantly compared with control cells. NaBT inhibited significantly the colony size, but knockdown of CLCA1 compromised NaBT-induced reduction of the colony size. Magnification of objective is 10×. 50 cysts were measured in each group in one experiment. **B.** Caco-2 cells were treated with negative control or CLCA1 siRNA and cultured for the days indicated. Cell proliferation was detected with EdU incorporation assay. EdU was visualized using Alexa Fluor 594 (Red) and DNA for DAPI (blue). The histogram presents the EdU positive % of cells in different groups and shows that knockdown of CLCA1 significantly increased the cell proliferation. *P* values are shown on the histogram. N = 100 in each group in one experiment. Scale bar = 100 µm in A, 50 µm in B. The results are shown as a mean±s.e.m from three independent experiments.

The antiproliferative effect of NaBT has been studied extensively in various malignant cell lines [25]. To verify whether CLCA1 presented an anti-proliferative effect on Caco-2 cells, we cultured cells in 3D Matrigel for 5 days to form colonies. We found that the mean colony size in Caco-2 CLCA1KD cells increased significantly compared with wild type cells (p<0.01, n = 50 each). When cells were treated with 2 mM NaBT, the colony size was inhibited significantly in Caco-2 cells, but in CLCA1KD cells with treatment of NaBT the colony size was not reduced significantly (*p*>0.05, n = 50 each) (Fig. 5A). These results indicate that the antiproliferative effect of NaBT could be mediated by CLCA1. To further confirm the effect of CLCA1 on proliferation, we assessed this via ethynyldeoxyuridine (EdU) incorporation in Caco-2 cells. We found that proliferation of Caco-2 cells was reduced in 3 day confluent cultures. However, proliferation of Caco-2 cells was promoted significantly in siRNA^clca1^ treated cells when compared to a siRNA negative control cells (*p*<0.01, n = 100 each) (Fig. 5B). Together with the 3D culture, these results show that the expression of CLCA1 contributes to the regulation of proliferation in Caco-2 cells.

## Discussion

The proliferation to differentiation transition (PDT) is a critical step in the continual renewal of a normal intestinal epithelium [1] and colon epithelial cells are amongst the best-studied models of tumorigenesis since their constant renewal requires close regulation of the PDT [3], [28]. Many genetic lesions, including mutation of APC and p53, produce neoplastic transformation of the colon enterocyte. In addition, during organogenesis, stem cells execute the silencing of proliferation genes and the activation of differentiation genes in a step-wise temporal manner. Important insights about the molecules that may serve as targets for therapy would be gained from a full understanding of the molecular mechanisms of these processes. Here, we found that the calcium activated chloride channel CLCA1 plays a crucial role in regulation and maintenance of PDT in colon enterocyte, since loss of CLCA1 led to reversion of cells to a low-differentiated status.

### CLCA1 Regulates the PDT in Intestinal Epithelial Cells

The PDT of single progenitor cell is tightly regulated by morphogens, growth factors and hormones [29] and molecular alterations to specific components of the signaling pathways used by these different classes of molecule are important during the development of cancer [30]. Of these changes, upregulation of CDK inhibitors (CKI) p21^Cip1/WAF1^ and p27^Kip1^ in many terminally differentiating cells [31], Wnt/β-catenin signaling inducing muscle cell differentiation [32], SOX9-dependent PKCα repression favoring proliferation and inhibiting differentiation [33] have been reported. Our previous study has demonstrated that CLC-2, CLC-4 (chloride channel 2 and 4) and CFTR (cystic fibrosis transmembrane regulator, a chloride channel) were expressed at significantly higher level in freshly isolated human corneal tissues than in cultured epithelial cells (primary culture or cell line) [34]. As the number of cell-layers increases, the gene expression level and protein staining intensity of CLCA2 (calcium-activated chloride channel member 2) was increased significantly [35]. This indicates that the CLCs (Chloride channels) may contribute to the regulation of differentiation in the development of epithelial tissues. In addition, the activity of Cl^−^ channels varies with the cell cycle and loss of calcium-activated chloride channels would afford a significant growth advantage to tumor cells [14], [15], [35], [36], [37]. In CRC, CLCA1 and CLCA2 was downregulated significantly in approximately 80% of patients [19]. The loss of expression of both mCLCA1 and mCLCA2 during tumorigenesis suggested that strong activation of either might inhibit survival of tumor cells [16]. CLCA2 is required for epithelial differentiation, and its loss during tumor progression contributes to metastasis [38]. Proliferating Caco-2 cells spontaneously initiate the differentiation process when the cells have reached confluence. This cellular differentiation program is initiated upon cell–cell contact and the switch from proliferation to differentiation may be triggered by specific biochemical events including E-cadherin mediated cell–cell adhesion [20]. Chloride channels are membrane protein and may play an important role in cell-cell communication after cell-cell adhesion. Collectively, this implies that the CLCAs might play a functional role in tumorigenesis through controlling the proliferation and differentiation balance.

The CLCA1 precursor is about 900 amino acids long with one proteolytic cleavage site following the amino-terminal signal sequence. Eventually, two products of a 90-kDa and a 30–40-kDa play functional roles [15], [16], [36], [37]. CLCA1 is closely correlated with transcription of c-*myc,* a proto-oncogene whose product is intimately involved in the regulation of cell proliferation and apoptosis [39]. In this study, we found using microarray that the expression of CLC2, CLC3, CLCA1, CLCA4 and CFTR were down-regulated significantly in early CRC patients and this reduced expression of CLCA1 was confirmed by immunofluorescent staining of tissue from CRC patients (Fig. 1). In addition, *in vitro* we showed that CLCA1 was involved in regulating the differentiation and proliferation of Caco-2 cells. When CLCA1 in Caco-2 cells was inhibited with siRNA^clca1^, both NaBT-induced and spontaneous differentiation was reduced significantly (Fig. 4), whereas proliferation increased significantly (Fig. 5). These data suggest that activation of CLCA1 may be crucial in maintaining the balance between differentiation and proliferation of enterocytes.

In addition, the high tissue specificity of transcription of some CLCs [40], initially suggested that detection of their mRNA in specific tissues might be useful for early diagnosis, molecular staging, and postoperative surveillance [19]. Importantly, our data show that inappropriate transcription of chloride channels not only included CLCA1, but also CLC2, CLC3, CLCA4 and CFTR, indicating that defects of chloride transport or of chloride current may play a key role in CRC. Activation of chloride current through specialized volume-regulated anion channels (VRACs) is one of the major mechanisms by which cells restore their volume following hypo-osmotic stress (RVD) [11], whilst the transepithelial transport of chloride also contributes to the transepithelial potential difference (TEP) in intestine [41]. The TEP is an inherent property of transporting epithelia and arises from gradients of ions transported directionally across epithelial cell layers. Across human intestine, there is a TEP of −25±7 mV, lumen negative. It will be interesting to test the novel notion that the TEP may play regulatory roles in controlling PDT and tumourgenesis of intestine.

### β-catenin is Involved in CLCA1-driven Cell Proliferation and Differentiation

The Wnt pathway is highly conserved throughout the animal world [4]. β-catenin is a central molecule in the canonical Wnt pathway that regulates intestinal epithelial differentiation [4], [42], [43]. In addition, the β-catenin/TCF (T-cell factor) pathway also regulates colonic epithelial cell proliferation [33]. In mouse myoblast cells C2C12, Wnt signaling via β-catenin may act as a molecular switch that regulates the transition from cell proliferation to myogenic differentiation [44]. In addition most instances of CRCs arise from inactivating mutations in the adenomatous polyposis coli (*APC*) gene, that controls β-catenin degradation [28], [45], [46]. Collectively, this suggests that the β-catenin pathway plays a key role in PDT. In Caco-2 confluent cultures, as they differentiate, β-catenin showed a time dependent increase on the cell membrane (Fig. 3) [27] and when CLCA1 was knocked down, β-catenin was down-regulated dramatically on the cell membrane (Fig. 4). Our data implies that β-catenin is involved in the cascade of events leading to differentiation and this is driven by CLCA1 activation in Caco-2 cells.

### CLCA1 as a Target of Butyrate in Pro-differentiation and Anti-proliferation

Butyrate is one of the most abundant short chain fatty acids (SCFAs) and plays a key role in colonic epithelium homeostasis. It is oxidized to acetyl CoA in mitochondria and represents the main fuel for normal enterocytes [47], [48], as well as for colon cancer cells [49]. In human colon cancer cells, butyrate inhibits cell growth [49], [50], [51] and promotes cell differentiation [52]. In addition, butyrate-induced differentiation of PC12 cells to chromaffin cells involves tight cell adhesion and the induction of extracellular cell adhesion proteins [53]. Anticarcinogenic effects of butyrate have been observed using carcinoma cell lines *in vitro*. In these models, butyrate leads to inhibition of proliferation, induction of apoptosis, or differentiation of tumor cells [54], [55], [56], [57]. Furthermore, many mechanisms of butyrate’s anticarcinogenic effects have been reported, for example it is a histone deacetylase inhibitor [58], [59], [60], [61], increases p21 (*WAF1*) gene expression and induces G1 cell cycle arrest [62]. Butyrate also down-regulates the key apoptotic and angiogenesis regulator neuropilin-1 (NRP-1) to inhibit tumor cell migration and survival in colon cancer [63]. In addition, butryrate dysregulates Bcl2 family proteins [64], [65], induces GPR109A expression and activation of the receptor to cause tumor cell–specific apoptosis [66], and modulates canonical Wnt signaling [67]. Butyrate also increases the differentiation of human LIM2537 colon cancer cells, decreases GSK-3β activity and increases levels of both membrane-bound and Apc/axin/GSK-3β complex-associated pools of β-catenin [39]. Butyrate is recognized for its potential to act on secondary chemoprevention [68]. Our results indicate that CLCA1 as a target of butyrate, effectively regulate pro-differentiation and anti-proliferation in Caco-2 cells (Fig. 2 and 5).

In summary, we reveal a novel mechanism that CLCA1 regulates the Wnt/β-catenin driven spontaneous and butyrate-induced differentiation of enterocytes. This indicates that CLCA1 may control PDT of enterocyte and therefore act as a tumour suppressor in colorectal tumorigenesis. Loss of CLCA1 expression inhibits enterocyte differentiation and may lead to colonic cancer development. Thus, better understanding of the CLCA1 phenotype in colonic epithelium and the mechanisms underlying loss of expression in carcinomas may provide a means of therapeutic intervention through reversal of progression of human colorectal carcinogenesis.

## Materials and Methods

### Ethics Statement

The colon and rectal tissues were obtained from surgery of patients in the Department of General Surgery, the 309th Hospital of PLA, Beijing, China. Ethical approval for the study was granted by the 309th Hospital of PLA Ethics Committee. Informed written consent was obtained from all participants involved in the study.

### Cell Culture

Caco-2 cells (ATCC, HTB-37) were cultured in Dulbecco’s Modified Eagle Medium (DMEM) (Invitrogen, UK), supplemented with 10% fetal bovine serum (FBS), 2 mM L-glutamine, non-essential amino acids, 50 U/ml penicillin and 50 µg/ml streptomycin at 37°C in a 5% CO2 incubator. Sodium butyrate was purchased from Sigma-Aldrich, UK.

### Knockdown of CLCA1 with siRNA

Knockdown of CLCA1 was performed using BLOCK-iT™ RNAi Express search engine (Invitrogen, UK). Stealth RNAi™ siRNA duplex with sense-strand sequences 5′- CAAUGCUACCCUGCCUCCAAUUACA-3′ was submitted in a BLAST search of human EST libraries to ensure that other human genes were not targeted. In brief, the cells were cultured in DMEM containing 10% FBS without antibiotics one day before transfection. Caco-2 cells then were transfected with CLCA1 specific siRNAs using lipofectamine 2000 diluted in Opti-MEM I medium according to manufacturer’s protocol (Invitrogen, UK) with a final siRNA concentration from 50–200 nM for optimization of siRNA transfection. Non-targeting negative control siRNA was used for non-sequence-specific effects of these molecules. For 10 day monolayer cultures, 5×10^4^ cells were plated into collagen I pre-coating coverslips or 12-well plates. 100 nM of siRNA duplex was transfected on the 2^nd^ and 6^th^ day of culture. After 10 days in culture, cells on cover slips were fixed and cells in 12-well plate were lysed for western blot analysis.

### Quantitative PCR

Caco-2 cells were cultured in DMEM with/without 2 mM NaBT treatment for 24 hours at 37°C with 5% CO_2_. Total RNA was isolated using TRIzol® Reagent (Invitrogen, UK). cDNA was synthesized with SuperScript™ III Cells Direct cDNA Synthesis System (Invitrogen, UK). Expression level of CLCA1, CLCA2, CLCA3 and CLCA4 mRNA were determined using quantitative real time polymerase chain reaction (qPCR). PCR primers were designed using Primer3 online [34]. Human CLCA1, CLCA2, CLCA3 and CLCA4 mRNA sequences were downloaded from the National Center for Biotechnology Information (NCBI) GenBank. Regions that matched the consensus sequence for CLCA1, CLCA2, CLCA3 and CLCA4 were chosen for PCR primers (Table 1). Primers were chosen to be 20 bp in length, with a GC content of 55% and no long repeats of a single base. 1∶5 diluted cDNA was used to run qPCR (SYBR green, Roche, Switzerland) on LightCycler® *480* System. All quantitative RT-PCR assays were carried out in duplicates and repeated with templates from 3 independent experiments, and analysed using Lightcycler software (Roche, Switzerland).

**Table 1:** PCR primers for detection of members of CLCA.

Gene name	Orientation	Start	Length	Tm	GC%	Sequence (5′->3′)	Product Size
CLCA1	FORWARD	433	20	59.99	50	GCTGATGTTCTGGTTGCTGA	199
	REVERSE	631	20	59.81	50	CGTCAAATACTCCCCATCGT	
CLCA2	FORWARD	1239	20	59.83	60	GATCAGAGCCCAGCTACACC	197
	REVERSE	1435	20	59.8	55	GCTTATCATCTCCGCTGGTC	
CLCA3	FORWARD	1849	20	59.99	60	GCCCTACCACACTCCCAGTA	182
	REVERSE	2030	20	60.07	50	ATTGTCCCAGAGCTCCAATG	
CLCA4	FORWARD	1731	20	59.96	50	GGCACTTGGGCATACAATCT	200
	REVERSE	1930	20	60.11	50	ACATTGGCTCCAAGAACAGG	

### Immunofluorescent Staining

The colon and rectal tissues were fixed with 4% paraformaldehyde in PBS for 2 hours. After paraffin embedding the tissue was cut into 10 µm thick sections and mounted on charged glass slides. Slides were de-paraffinized and subjected to citrate-based antigen retrieval. Tissue sections were blocked with PBS containing 10% goat serum, 2% bovine serum albumin (BSA) plus 0.3% Triton X-100 for one hour at room temperature to reduce non-specific antigen binding. Rabbit anti-CLCA1 (1∶100, Santa-Cruz, USA) was applied overnight at 4°C in a moist chamber. After washing with PBS, the tissue sections were incubated with Alexa Fluor® 555 highly cross-adsorbed secondary antibodies (Invitrogen, UK) and phalloidin-FITC (Sigma–Aldrich, UK) for 1 hour at room temperature. For monolayer staining, cells were fixed in 4% paraformaldehyde for 20 minutes, permeabilized for 5 minutes, blocked for 30 minutes and followed by 2 hours incubation with β-catenin (1∶100, Cell Signaling, USA) antibodies. After washing with PBS, cells were incubated with Alexa Fluor® 488 highly cross-adsorbed secondary antibodies (Invitrogen) for 1 hour. Both colonic tissue and Caco-2 monolayers were mounted in Vector mounting medium with DAPI (Vector Labs, UK) and sealed under coverglass with clear nail polish. Images were obtained with the Zeiss Axio Observer Z1 inverted fluorescence microscope for tissue and with a confocal 700 LSM (Carl Zeiss, Germany) for monolayers.

### Western Blotting

5×10^5^ Caco-2 Cells were cultured on collagen I pre-coated 6-well plates with different treatments for the indicated time. Cells were rinsed with cold PBS and lysed with cell lysis buffer (Cell signaling, USA) and a protease inhibitor cocktail (Roche, Switzerland). Concentration of proteins was assessed with Bradford assay (Pierce, USA). Equal amounts of protein lysates were resolved by 4–12% SDS-PAGE, followed by electroblot analysis onto nitrocellulose membranes. The membranes were stained with Ponceau S for detection of transfer efficiency, then were blocked with 5% milk TBS with 0.1% (w/v) Tween 20 for 1 hour. Primary antibodies used for WB included anti-CLCA1 (1∶1000), anti-sucrase-isomaltase (1∶400) (Santa Cruz, USA), anti-ALPI (1∶2000 NOVUS, USA), anti-β-catenin (1∶3000, cell signaling, USA) and anti-GAPDH (1∶50,000 Santa Cruz, USA). Membranes were incubated with the relevant primary antibodies overnight at 4°C. The secondary antibody with horseradish peroxidase (1∶5000, Sigma-Aldrich, UK) was used, and the immunoblots were detected by Luminata Forte Western HRP substrate (Millipore).

### Microarray Data Mining and Analysis

The microarray data sources were from the Gene Expression Omnibus (GEO) [69]. Data sets were not subjected to any additional normalization, as all had been normalized when we obtained them [70]. Tumor specimens and adjacent grossly normal-appearing tissue at least 8 cm away were routinely collected and archived from patients undergoing colorectal resection at the Singapore General Hospital (n = 12 from tumour specimens, n = 10 from normal tissue). All patients, except one, had early stage (Dukes A/B) tumors at the time of the operation. Affymetrix Human Genome U133 Plus 2.0 Array (Affymetrix, CA) was used for Genome-wide expression analysis [70]. We filtered the GDS2609 data set for the expression of chloride channel family members using the software on GEO. The identity of genes across microarray data sets was established using public annotations, primarily based on Unigene [71].

### Differentiation and Proliferation Assays

Cellular differentiation of Caco-2 cells was measured after 72 hrs culture with either 100 nM negative control or CLCA1 siRNA treatment. Alkaline phosphatase activity was determined using the Alkaline Phosphatase Detection Kit (BioVision) according to manufacturer’s specifications. Fluorescence intensity at Ex/Em 360/440 nm was recorded using a fluorescence microtiter plate reader (Perkin-Elmer). All experiments were repeated at least 3 times and all measurements were performed in triplicate.

Cell proliferation was assessed by EdU (5-ethynyl-2′-deoxyuridine) incorporation into DNA with a Click-iT® EdU kit (Invitrogen). In brief, Caco-2 cells were cultured for the indicated time and then were incubated with 10 µM of EdU for 2–4 h. The cells were fixed with 4% paraformaldehyde in PBS and then were detected for EdU staining following the manufactures instructions.

### 3D Culture Assay

The establishment of tumour cell 3D cultures was performed as previously described [72]. Briefly, Caco-2 cells were seeded on top of the solidified Matrigel (BD) layer in each well of an 8-well chamber slide and incubated for 5 days to allow for multicellular colonies to form. Phase contrast images were taken under light microscopy. Images were analyzed using Metamorph software 7.0. Colony formation as well as colony size reflects the number of cell and tumour cell proliferation status.

### Statistics

A minimum of three replicates was analyzed for each experiment presented. Data are presented as the mean ± s.e.m. Student’s t test was used to assess the statistical significance. Differences were considered as significant with a *P* value <0.05.
